# Endocytosis of Albumin by Podocytes Elicits an Inflammatory Response and Induces Apoptotic Cell Death

**DOI:** 10.1371/journal.pone.0054817

**Published:** 2013-01-28

**Authors:** Kayo Okamura, Patrick Dummer, Jeffrey Kopp, Liru Qiu, Moshe Levi, Sarah Faubel, Judith Blaine

**Affiliations:** 1 Department of Medicine, University of Colorado Denver, Aurora, Colorado, United States of America; 2 Kidney Disease Section, National Institute of Diabetes and Digestive and Kidney Diseases, National Institutes of Health, Bethesda, Maryland, United States of America; National Cancer Institute, United States of America

## Abstract

The presence of albuminuria is strongly associated with progression of chronic kidney disease. While albuminuria has been shown to injure renal proximal tubular cells, the effects of albumin on podocytes have been less well studied. We have addressed the hypothesis that exposure of podocytes to albumin initiates an injury response. We studied transformed human-urine derived podocytes-like epithelial cells (HUPECS, or podocytes). Upon differentiation, these cells retain certain characteristics of differentiated podocytes, including expression of synaptopodin, CD2AP, and nestin. We exposed podocytes to recombinant human albumin, which lacks lipids and proteins that bind serum albumin; this reagent allowed a direct examination of the effects of albumin. Podocytes endocytosed fluoresceinated albumin and this process was inhibited at 4°C, suggesting an energy-dependent process. Exposure to albumin at concentrations of 5 and 10 mg/ml was associated with increased cell death in a dose-dependent manner. The mechanism of cell death may involve apoptosis, as caspase 3/7 were activated and the pan-caspase inhibitor z-VAD reduced cell death. Albumin exposure also increased nuclear factor (NF)-κB activation and increased transcription and release of interleukin (IL-) 1β, tumor necrosis factor (TNF), and IL-6. We extended these findings to an *in vivo* model. Glomeruli isolated from mice with nephrotic syndrome also had increased expression of IL-1β and TNF RNA. These data suggest that while podocyte injury begets albuminuria, albumin in the glomerular ultrafiltrate may also beget podocyte injury. Thus, an additional mechanism by which anti-proteinuric therapies are beneficial in the treatment of glomerular diseases may be a reduction in injury to the podocyte by albumin.

## Introduction

Albuminuria, one of the hallmarks of chronic kidney disease, is strongly and independently associated with kidney disease progression and cardiovascular events [1], [2], [3], [4], [5], [6]. While albuminuria has traditionally been defined as excretion of >30 mg/day albumin in the urine, recent studies have shown that excretion of as little as 5–10 mg/day of albumin is associated with an increased risk of progressive kidney disease and cardiovascular mortality [7]. Despite the strong association between albuminuria and progressive loss of kidney function, the effects of albumin at the molecular level on renal cells remains to be fully determined.

While the precise mechanisms governing how albumin is excreted into the urine are a matter of controversy and active investigation [8], [9], it is generally agreed that in individuals with normal renal function relatively little albumin traverses the glomerular filtration barrier (GFB) [10], [11]. The GFB consists of fenestrated endothelial cells, the glomerular basement membrane and podocytes and it is thought that size and charge constraints prevent the passage of albumin through this barrier [12]. In proteinuric states, however, significant quantities of albumin are found in the glomerular ultrafiltrate. In both humans and animals with heavy proteinuria podocytes have been shown to take up albumin. Protein resorption droplets have been found within podocytes in kidney biopsy specimens from patients and animals with nephrotic syndrome [13] and podocytes have been shown to endocytose albumin in cell culture [14].

The effects of albumin exposure on podocytes are not well understood. While some studies have reported deleterious effects of albumin exposure on cultured podocytes these studies used mouse podocytes and relatively high doses of bovine serum albumin (10–40 mg/ml) without any controls for the oncotic effects of added albumin [15], [16]. Here we use human urine derived podocyte-like epithelial cells (HUPECs or podocytes), transformed with human telomerase reverse transcriptase and temperature-sensitive SV40 antigen [17] to investigate the effects of albumin exposure on podocytes. Podocytes were cultured under growth-restricted conditions (37°C) at which they express differentiation markers including (WT1), synaptopodin and nestin. We exposed podocytes to a concentration of albumin (5 and 10 mg/ml) similar to that found in the urine of patients with nephrotic syndrome and used dextran of a similar molecular weight to albumin as an oncotic control. In addition, we confirmed some of our *in vitro* findings using a mouse albumin overload model [18] as an *in vivo* model of nephrotic syndrome.

## Materials and Methods

### Reagents

Human albumin conjugated to fluorescein isothiocyanate (FITC-albumin) was purchased from Cappel Laboratories (Malvern, PA). Recombinant, low-endotoxin human albumin was purchased from Sigma (St. Louis, MO). This albumin is fatty acid and globulin free. We confirmed using the Limulus Amebocyte Lysate assay (Lonza, Walkersville, MD) that the levels of endotoxin in this reagent are 0.0005 EU endotoxin/µg albumin (considered endotoxin-free by industry standards). Interleukin-6 (IL-6), tumor necrosis factor (TNF) and IL-1β ELISA kits were purchased from Biolegend (San Diego, CA). RNA was isolated using the RNeasy Mini Kit (Qiagen, Valencia CA). Rabbit polyclonal NFκB and IκB antibodies were purchased from Cell Signaling (Beverly, MA). Alexa 488 goat anti-rabbit secondary antibody was from Molecular Probes (Invitrogen, Carlsbad CA).

### Ethics Statement

Animal studies were performed under a protocol approved by the Institutional Animal Care and Use Committee at the University of Colorado. Sodium pentobarbital (60 mg/kg) was used to deeply anesthetize mice prior to perfusion and every effort was made to minimize suffering.

Human urine derived podocyte-like epithelial cells (HUPECs or podocytes) were isolated from the urine of patients who gave written informed consent under a protocol approved by the National Institute of Diabetes and Digestive and Kidney Diseases Institutional Review Board (National Institutes of Health, Bethesda, MD).

Human podocytes, initially characterized by Mojn Saleem, were isolated from a nephrectomy specimen as previously described. The specimen was obtained under the approval of the local ethics committee (University of Bristol, Bristol, UK).

### Cell Culture

#### HUPECS

Human urine derived podocyte-like epithelial cells (HUPECs, termed podocytes for simplicity) isolated from a healthy volunteer were described previously [17]. Cells were maintained in an undifferentiated state at 33°C in RPMI medium (Invitrogen) supplemented with 10% fetal bovine serum (FBS), insulin-selenium-transferrin G (Invitrogen) and penicillin streptomycin (Invitrogen). Medium supplemented with FBS, insulin-transferrin and penicillin streptomycin is hereafter referred to as complete medium. Since FBS contains 23 mg/ml albumin complete medium had a concentration of 2.3 mg/ml albumin. Prior to each experiment podocytes were plated in collagen-coated dishes and placed at 37°C for 10–14 days to allow differentiation. Under these conditions cells express podocyte markers including synaptopodin, nestin and CD2AP.

#### Human Podocytes from Moin Saleem

A previously described immortalized human podocyte line [19] was a kind gift of Dr Moin Saleem (University of Bristol). Podocytes were maintained in an undifferentiated state at 33°C in complete medium. Prior to use in experiments, podocytes were plates in collagen coated dishes and allowed to differentiate at 37°C for 9–12 days.

### Animals

7 week old female Balb/c mice were obtained from Jackson Laboratories (Bar Harbor, MA). Animals were given free access to food and water and studies were performed in accordance with the guidelines of the Institutional Animal Care and Use Committee of the University of Colorado.

### Albumin Uptake

Podocytes were plated at a density of 2.8×10^4^ cells per dish in 35 mm collagen coated dishes (MatTek Corporation, Ashland, MA) and allowed to differentiate for 10–14 days. After differentiation, cells were placed at 4°C (inhibits endocytosis) or 37°C (promotes endocytosis), washed once with phosphate buffered saline and incubated in Ringers solution (in mM: 122.5 NaCl, 5.4 KCl, 1.2 CaCl_2_, 0.8 MgCl_2_, 0.8 Na_2_HPO4, 0.2 NaH_2_PO4, 5.5 glucose, and 10 HEPES, pH 7.4,) for 2 hrs. Podocytes were treated with 1.5 mg/ml human albumin coupled to fluorescein-isothiocyanate (FITC), in Ringers. After incubation with FITC-albumin, podocytes were washedextensively with ice cold PBS and scraped into 20 mM MOPS +0.1% Triton-X 100. Cell associated fluorescence was analyzed on an ISS Spectrofluorometer (Champaign, IL) using an excitation wavelength of 490 nm and an emission wavelength of 540 nm. Results were normalized to total protein content, which was measured using the bicinchoninic acid (BCA) assay (Pierce, Rockford IL). All experiments were performed in triplicate.

For imaging of albumin uptake, podocytes were placed at 4°C or 37°C, washed once with PBS and pre-incubated in Ringers solution as described above. Cells were treated with 1.5 mg/ml human FITC-albumin in Ringers for 1 hr. Cells were washed very well in ice-cold PBS, fixed in 4% paraformaldehyde, and imaged using a confocal microscope.

### Trypan Blue Exclusion Assay

The cells to be counted were trypsinized and collected in a microfuge tube. The cellular suspension was centrifuged at 1000*g* for 5 minutes, the pellet was washed once with phosphate buffered saline and resuspended in a small volume of PBS. An aliquot of cell suspension was transferred to a 96-well plate and incubated for 3 minutes at room temperature with an equal volume of 0.4% trypan blue solution [20]. The cells were counted using a hemocytometer under a light microscope. Viable and nonviable cells were recorded separately. All experiments were performed in triplicate.

### Caspase 3/7 Activity

Podocytes were plated in 24 well plates and allowed to differentiate for 10–14 days. Cells were treated with 5 mg/ml low endotoxin recombinant human albumin or dextran of an equivalent molecular weight to albumin for varying lengths of time. At the time of harvest podocytes were rinsed once with ice-cold PBS and lysed in radio-immunopreciptation (RIPA) buffer with Complete protease inhibitors added (Roche). Caspase activity was determined using the Caspase 3/7 Activity kit (AAT Bioquest, Sunnyvale, CA) according to the manufacturer’s directions. For each condition caspase amount was normalized to the cellular protein content. All experiments were performed in triplicate.

For the benzyloxycarbonyl-Val-Ala-Asp fluoromethylketone (z-VAD) inhibition experiments, z-VAD was dissolved in dimethyl sulfoxide (DMSO) to make a stock solution of 100 mM. Podocytes were treated with 5 mg/ml recombinant human albumin +50 µM z-VAD or 5 mg/ml albumin+DMSO at a concentration of 1∶2000.

#### Terminal deoxynucleotidyl transferase dUTP nick end labeling (TUNEL) assay

Podocytes were plated at a density of 2.8×10^4^ cells per dish in collagen coated 35 mmdishes with coverslip bottoms (MatTek, Ashland, PA). Cells were treated with 5 mg/ml low endotoxin human albumin or dextran of an equivalent molecular weight to albumin in complete medium for 24 or 48 hrs. The TUNEL assay was performed using the ApoAlert DNA Fragmentation Assay kit (Clontech, Mountain View, CA). Nuclei were examined for TUNEL staining using a confocal microscope.

### NFκB Nuclear Translocation

Podocytes were plated in MatTek dishes as described above. Cells were treated with 5 mg/ml low endotoxin human albumin or dextran of an equivalent molecular weight to albumin in complete medium. After treatment, podocytes were fixed in 4% paraformaldehyde, washed and permeabilized in PBS-X (PBS +0.1% Triton-X 100). Cells were blocked for 30 minutes in blocking solution (PBS-X +5% goat serum) and rabbit anti-NFκB antibody at a dilution of 1∶100 was applied overnight. The following day, cells were washed 3 times in PBS-X and goat-anti rabbit Alexa 488 secondary antibody at a dilution of 1∶250 was applied. After 4 additional washes cells were mounted in Vectashield+DAPI (Vector Laboratories, Burlingame, CA).

### RNA Preparation, cDNA Synthesis and Real-Time PCR

For cultured podocytes total RNA was isolated using RNeasy Mini Kit (Qiagen) according to the manufacturer’s instructions. cDNA was synthesized from total RNA (1 µg) with the High Capacity cDNA Reverse Transcriptase Kit (Invitrogen) which uses the random primer scheme. The primers used for IL-6, TNF, IL-1β, and VEGF are as follows: IL-6 sense 5′-ATG AAC TCC TTC TCC ACA AGC GC-3′, antisense 5′-GAA GAG CCC TCA GGC TGG ACT G-3′; TNF sense 5′-CAA TGG CGT GGA GCT GAG AGA-3′, antisense 5′-CCA AAG TAG ACC TGC CCA GAC-3′; IL-1β sense 5′-AAC AGG CTG CTC TGG GAT TCT CTT-3′ antisense 5′-ATT TCA CTG GCG AGC TCA GGT ACT-3′; VEGF sense 5′-CCC TGA TGA GAT CGA GTA CAT CTT-3′, antisense 5′-AGC AAG GCC CAC AGG GAT TT-3′. Real-time PCR was performed with the use of the AppIied Biosystems Step One Plus Real-Time PCR System (Life Technologies, Carlsbad, CA).The expression of a target gene in relation to a reference gene was calculated using a comparative cycle threshold (Ct) method. The results are given as relative expression normalized to the 18S gene. All experiments were performed in triplicate.

### Western Blot Analysis

Podocytes were grown in 35 mm collagen coated MatTek dishes and allowed to differentiate for 10–14 days. After differentiation cells were treated with 5 mg/ml recombinant human albumin or dextran for varying lengths of time. At the time of harvest podocytes were solubilized in RIPA buffer containing Mini-Complete protease inhibitors and centrifuged at 12000 *g* at 4°C for 20 minutes. Protein concentrations were determined using the BCA assay (Pierce). 20 ug of each sample was resolved by SDS-PAGE under reducing conditions and transferred to nitrocellulose membranes. The membranes were blocked with blocking buffer (5% milk in 1X PBS plus 0.1% Tween) for one hour at room temperature and incubated with IκB antibody (Cell Signaling) overnight at 4°C, followed by incubation with HRP-conjugated secondary antibody for 1 hr. Immunoreactive proteins were detected by enhanced chemiluminescence (Super Signal West Pico, Pierce). Alpha-tubulin was used as a loading control.

### ELISA

Podocytes were plated at a density of 2.8×10^4^ cells in 35 mm collagen coated dishes and allowed to differentiate for 10–14 days. Cells were treated with 5 mg/ml low endotoxin recombinant human albumin or 5 mg/ml dextran of an equivalent molecular weight to albumin, added to complete medium. At the time of harvest, the cell supernatant was collected and the amount of cytokine present was analyzed using IL-6, TNF, or Il-1β ELISA kits (Biolegend, San Diego, CA) The podocytes in each dish were harvested in ice-cold (RIPA) buffer (50 mM Tris HCl pH 8, 150 mM NaCl, 1% NP-40, 0.5% sodium deoxycholate, 0.1% SDS) with Mini-Complete protease inhibitors added (Roche, Indianapolis, IN). Cytokine amount was normalized to total protein content which was determined using the BCA assay. All experiments were performed in triplicate.

### In vivo Albumin Overload Model

7 week old female Balb/c mice were obtained from Jackson Laboratories (Bar Harbor, MA). Animals were given free access to food and water. Albumin overload was performed as described by Chang et al [18]. Briefly, mice were given daily intraperitoneal injections of fatty acid free low endotoxin bovine serum albumin (BSA, Sigma) dissolved in sterile saline starting at 2 mg BSA/gm body weight and increasing by 2 mg/gm body weight a day for 5 days until a final dose of 10 mg/gm body weight was reached. No injections were given on days 6 and 7 and then a final injection of a dose of 10 mg BSA/gm body weight was given on day 8. Mice were sacrificed on day 9. Saline injected controls received an equal volume of saline only. Mice were placed in individual metabolic cages on days 4 and 5 and urine collected for later analysis. The amount of protein in each urine sample was measured using the Bradford assay (Sigma). Urinary creatinine was measured using the QuantiChrom Creatinine Assay Kit from BioAssay Systems (Hayward, CA).

### Isolation of Mouse Glomeruli

Glomeruli were isolated using a modified procedure of Takemoto et al. [21]. Briefly, mice were anesthetized by intraperitoneal injection of pentobarbital sodium solution (2 mg/g mice body weight) and perfused with 20 ml of iron oxide solution in 1X PBS through the heart. The kidneys were removed, minced into pieces in Hanks buffered saline solution (HBSS), and gently pressed through a 100 µm cell strainer using a flattened pestle. The filtered cells were passed through a new cell strainer and the strainer was washed once with HBSS. The cell suspension was transferred to a 1.5 ml eppendorf tube, and the tube was placed in a rack containing a powerful magnet, allowing the iron oxide-containing glomeruli to stick to the wall of the tube. The accumulated glomeruli were washed twice with HBSS. Isolated glomeruli were immediately frozen in liquid nitrogen and kept at −80°C until use.

For isolated mouse glomeruli total RNA was isolated from glomeruli using the Quick-RNA MiniPrep (Zymo Research, Irvine, CA) according to the manufacturer’s directions. Total RNA concentration was determined spectrophotometrically at 260 and 280 nm. Possible genomic DNA contamination was removed by treatment with DNase I (Sigma) before RT-PCR was performed. The total RNA was reverse transcribed and real-time PCR was performed as described above with the exception that the primers used were as follows: IL-6 sense CTG GAA GAG ACT TCC ATC GAG TT; IL-6 antisense GAA GTA GGG AAG GCC GTG G; TNF sense CTG AAC TTC GGG GTG ATC GG; TNF antisense GGC TTG TCA CTC GAA TTT TGA; IL-1β sense AGA AGC TGT GGC AGC TAC CTG; IL-1β antisense GGA AAA GAA GGT GCT CAT GTC C.

### Confocal Microscopy

Confocal images were acquired using a Zeiss 510 laser scanning confocal microscope (Carl Zeiss, Thornwood, NY). FITC-albumin and Alexa 488 secondary antibodies were excited using a 488 nm laser.

### Data Analysis

Data are presented as mean ± SD. Differences between groups were analyzed using a two-tailed t-test for 2 groups or a one-way ANOVA with Dunnett’s post test for more than 2 groups. P<0.05 was taken to be statistically significant.

## Results

### Human Podocytes Endocytosed Albumin

**Figure 1:**
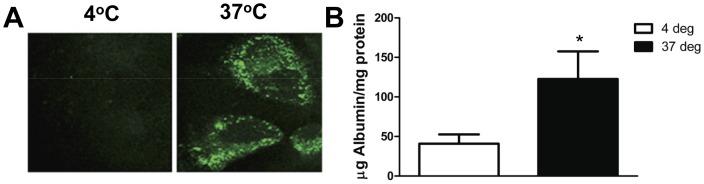
Cultured human podocytes endocytose albumin. *A*, Confocal image of cultured human podocytes placed at 4°C (inhibits endocytosis) or 37°C (promotes endocytosis) and treated with 1.5 mg/ml human FITC-albumin. Bright green vesicles seen in the podocytes at 37°C are FITC-albumin containing endosomes. *B,* Podocytes were treated with 1.5 mg/ml FITC-albumin at 4°C or 37°C for 2 hrs. There is a significant increase in the amount of albumin taken up by podocytes at 37°C compared to those at 4°C. * denotes P = 0.01 compared to albumin uptake at 4°C.

Podocytes were treated with 1.5 mg/ml human FITC-albumin and placed at 4°C (inhibits endocytosis) or 37°C (promotes endocytosis). Albumin vesicles were seen within podocytes placed at 37°C but not at 4°C (Fig. 1A). After 2 hrs of albumin exposure there was a significant increase in the amount of albumin taken up by podocytes at 37°C compared to 4°C (Fig. 1B). The amount of FITC-albumin normalized to total cellular protein at 2 hrs at 37°C was 122.5±15.6 µg albumin/mg protein compared to 40.8±5.2 µg albumin/mg protein for cells placed at 4°C (P = 0.006). These results are consistent with an energy-dependent, active process of albumin uptake in podocytes such as receptor-mediated uptake.

### Albumin Exposure Increased Podocyte Death

**Figure 2:**
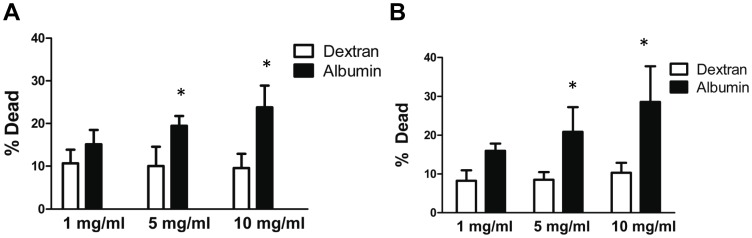
Albumin exposure increases cell death in podocytes. *A*, Cell death was measured using the trypan blue exclusion assay after treatment of podocytes with increasing doses of recombinant low-endotoxin human albumin (closed bars) or dextran of an equivalent molecular weight to albumin (open bars) for 24 hrs. * denotes P = 0.0003 compared to corresponding dextran-treated cells. *B*, Cell death after treatment of podocytes with increasing doses of human albumin (closed bars) or dextran (open bars) for 48 hrs. * denotes P<0.0001 compared to corresponding dextran-treated cells.

In order to examine whether albumin has deleterious effects podocytes, cells were treated with low endotoxin recombinant human albumin or dextran of an equivalent molecular weight to albumin (as an oncotic control) for 24 or 48 hrs. Albumin exposure (5 and 10 mg/ml) caused an increase in cell death (as assessed by the trypan blue exclusion assay) at both 24 hrs and 48 hrs (Figs. 2 A, B). Based on these data, we selected an albumin concentration of 5 mg/ml for subsequent experiments.

### Albumin Exposure Upregulated Pro-apoptotic Pathways

**Figure 3:**
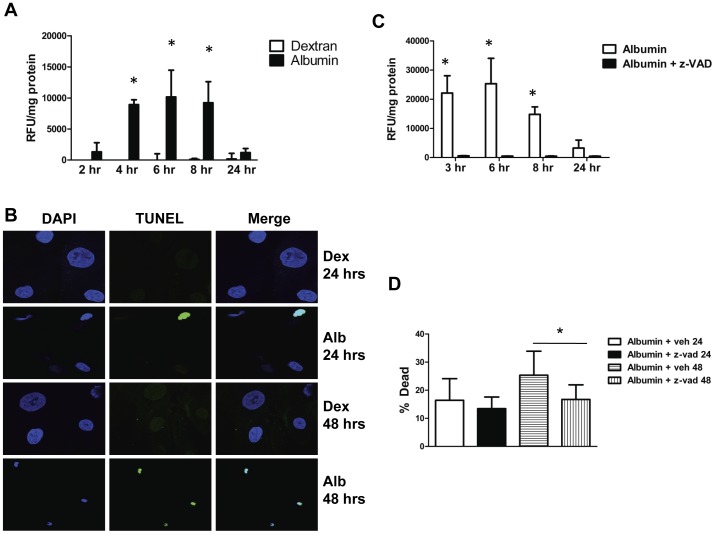
Albumin exposure upregulates pro-apoptotic pathways in podocytes. *A*, Activated caspase 3/7 activity normalized to total cellular protein in podocytes treated with 5 mg/ml albumin (closed bars) or 5 mg/ml dextran (open bars) as an oncotic control for the indicated amounts of time. * denotes P<0.0001 compared to corresponding dextran treated control. *B*, TUNEL staining of cultured podocytes treated with 5 mg/ml dextran (Dex) or 5 mg/ml albumin (Alb). Nuclei are stained blue with DAPI. TUNEL-positive nuclei are green. *C,* Caspase 3/7 activity normalized to total cellular protein in podocytes treated with 5 mg/ml albumin or 5 mg/ml albumin +50 µM z-VAD, a pan-caspase inhibitor. z-VAD largely abrogated caspase activity (*, P = 0.01 versus albumin+z-VAD). *D*, Percentage of dead cells measured using the trypan blue assay in podocytes treated with 5 mg/ml albumin alone (open bar) or 5 mg/ml albumin +50 µM z-VAD (closed bar) for 24 hrs or 5 mg/ml albumin alone (horizontal hatched bar) or 5 mg/ml albumin +50 µM z-VAD for 48 hrs (vertical hatched bar). * denotes P = 0.001 compared to albumin+z-VAD at 48 hrs.

We hypothesized apoptosis as one of the mechanisms underlying the increased cell death following albumin exposure. Caspases 3 and 7 are the final effector caspases in the apoptotic pathway [22], and we therefore examined caspase 3/7 activity in response to albumin. There was a significant increase in caspase 3/7 activity in albumin-treated cells that was not seen in dextran-treated cells (Fig. 3A). The increase in caspase 3/7 activity was relatively rapid, beginning 2 hrs after albumin treatment. Caspase 3/7 activity peaked between 6 and 8 hrs at 10,000-fold over control, and had abated by 24 hrs.

As further confirmation of increased apoptosis in albumin treated cells a TUNEL assay was performed on podocytes treated with 5 mg/ml albumin or dextran for 24 or 48 hrs. As can be seen in Fig. 3B podocytes treated with albumin an increase in TUNEL-positive nuclei versus control. TUNEL staining displayed a time-dependent relationship to albumin treatment, with a greater number of positive nuclei at 48 hours compared to 24 hours.

### Caspase Inhibition Decreased Podocyte Death after Albumin Exposure

Since albumin exposure upregulated pro-apoptotic pathways in podocytes we sought to determine whether inhibition of apoptosis would decrease cell death in response to albumin. Benzyloxycarbonyl-Val-Ala-Asp fluoromethylketone (z-VAD) is a cell-permeable potent pan-caspase inhibitor [23]. As shown in Fig. 3C, z-VAD suppressed caspase 3/7 activity in response to albumin treatment. To determine whether caspase inhibition decreased podocyte death in response to albumin we treated cells with 5 mg/ml albumin +50 µM z-VAD or 5 mg/ml albumin+vehicle and determined cell death using the trypan blue exclusion assay (Fig. 3D). After 24 hours of z-VAD treatment there was a trend towards decreased cell death, which reached significance at 48 hrs. The percentage of dead cells in the albumin+vehicle group at 48 hrs was 25.3±8.5% compared to 16.7±5.2% in the albumin+z-VAD group (P = 0.001).

### Albumin Exposure Increased Inflammatory Cytokine Expression and Release

**Figure 4:**
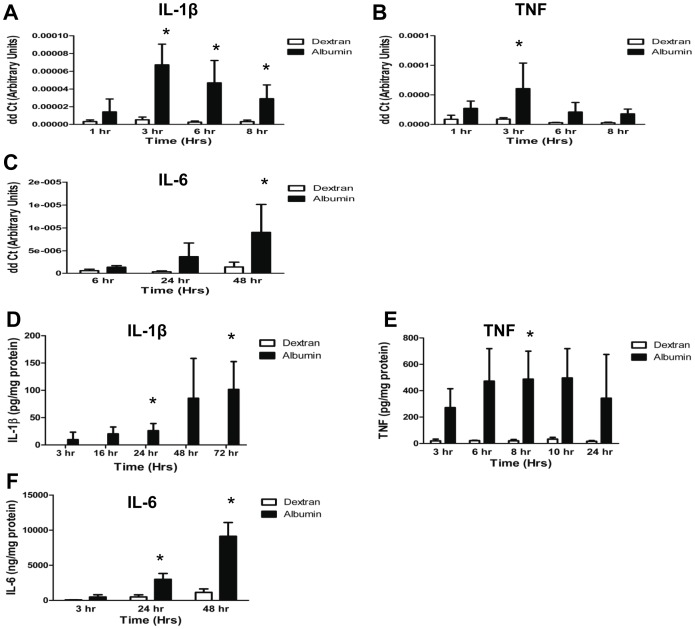
Albumin exposure modulates pro-inflammatory cytokine expression and release in human podocytes. *A*, Time course of IL-1β RNA levels in podocytes treated with 5 mg/ml recombinant low endotoxin human albumin (closed bars) or 5 mg/ml dextran (open bars). * denotes P = 0.0002 compared to dextran controls. *B*, Time course of TNF RNA expression in podocytes treated with 5 mg/ml human albumin (closed bars) or 5 mg/ml dextran (open bars). * denotes P = 0.01 compared to dextran control. *C*, IL-6 RNA levels in podocytes treated with 5 mg/ml human albumin (closed bars) or 5 mg/ml dextran (open bars). * denotes P = 0.008 compared to dextran control. D, Amount of IL-1β normalized to total cellular protein released into the medium after treatment of podocytes with 5 mg/ml recombinant human albumin (closed bars) or 5 mg/ml dextran (open bars) for varying amounts of time. * denotes P = 0.005 compared to dextran treated controls. *E*, Amount of TNF normalized to total cellular protein released into the medium by podocytes after treatment with albumin (closed bars) or dextran (open bars) for varying amounts of time. * denotes P = 0.003 compared to dextran treated control cells. *F*, Levels of IL-6 normalized to total cellular protein released into the medium by podocytes treated with albumin (closed bars) or dextran (open bars) for varying amounts of time. * denotes P<0.0001 compared to dextran treated controls.

Albumin exposure up-regulates inflammatory cytokine expression and release in renal proximal tubule cells but this has not been extensively studied in podocytes. We therefore sought to test whether albumin uptake in podocytes induced a similar inflammatory response. As shown in Figure 4A, podocytes treated with albumin had a significant increase in IL-1β RNA levels (P = 0.0002 versus dextran; Fig. 4A) compared to dextran treated controls. Albumin also significantly up-regulated TNF RNA expression at 3 hrs compared to dextran treated cells (P = 0.01 versus dextran; Fig. 4B). After 48 hrs of albumin treatment IL-6 RNA levels were also significantly increased (P = 0.008 versus dextran, Fig. 4A).

We sought to further confirm our findings by testing whether the increase in cytokine RNA expression was paralleled by increased cytokine release. Podocytes treated with albumin released more IL-1β, TNF, and IL-6 as compared to dextran treated cells as measured by ELISA (Fig. 4D, E, and F, respectively). Dextran-treated control podocytes displayed no change in IL-1β, TNF, or IL-6 at any time points. In contrast, albumin-treated podocytes displayed an increase in IL-1β beginning at 24 hrs of treatment (Fig. 4D). By 72 hrs the mean concentration of IL-1β released into the medium was 101.6±50.1 ng IL-1β/mg protein and below detection limits in control (P = 0.005). Significant TNF release appeared at 3 hours (Fig. 4E), with a 13.9 fold increase over dextran control (272±143.3 pg/mg and 19.6±13.3 pg/mg, respectively). The concentration of TNF peaked at 8 hours, with a 23.8 fold increase over dextran control (487.5±212.3 pg/mg versus 20.5±10.1 pg/mg, P = 0.003) and remained elevated 24 hours after treatment. Albumin exposure also increased IL-6 production in cultured podocytes (Fig. 4 F). The mean concentration of IL-6 released into the medium by podocytes exposed to 5 mg/ml albumin for 24 hrs was 6.1 fold greater than control (2998±837.5 ng/mg versus 492.9±296.9 ng/mg, P<0.0001), and continued to increase after 48 hours of treatment (9115±1138 ng/mg versus 1135±492 ng/mg, P<0.0001). This phenotypic change resulting from albumin exposure is in accord with induction of inflammatory response.

### Albumin Exposure Activates NFκB

**Figure 5:**
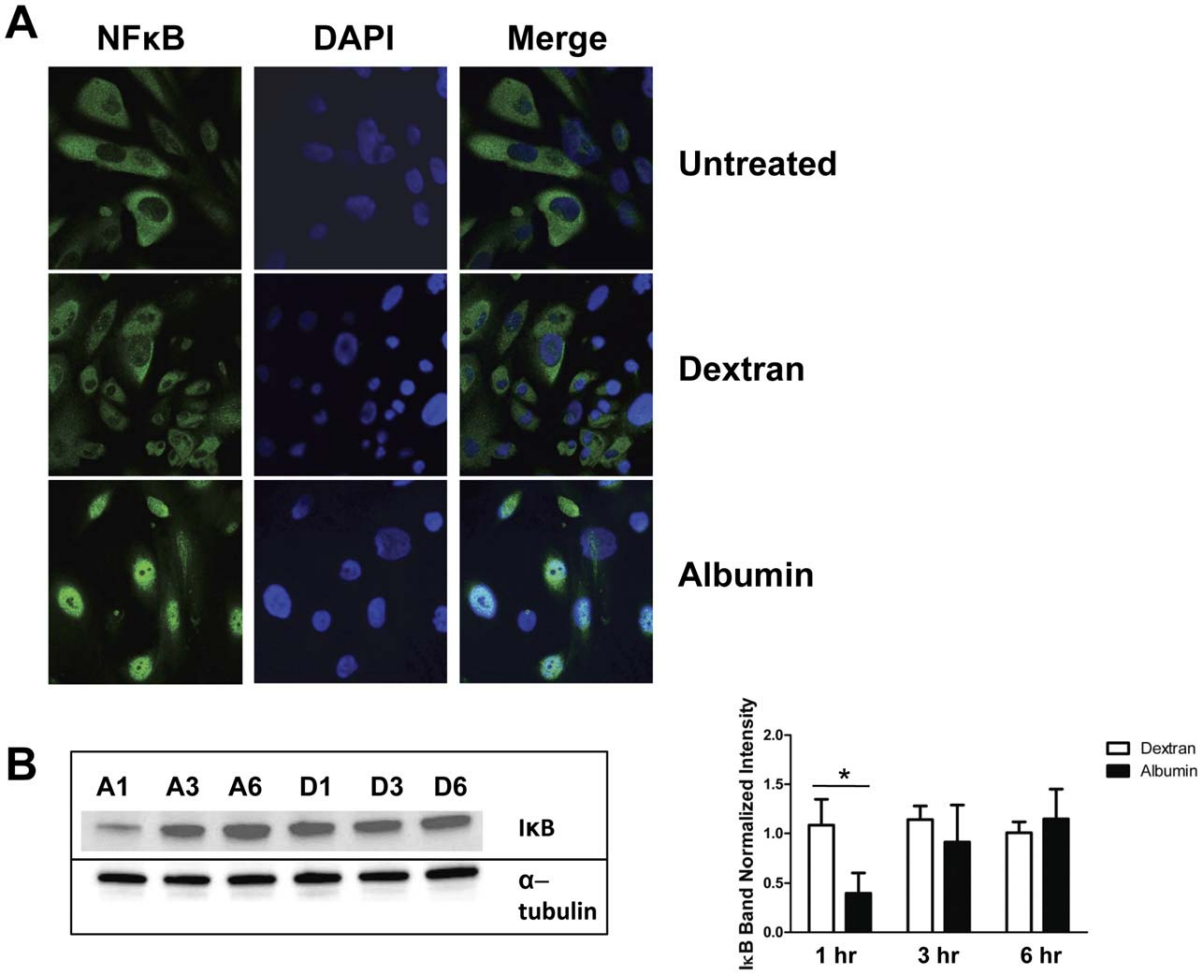
Albumin exposure activates NFκB. *A*, Confocal images of cultured human podocytes without treatment (top panel), and treated with 5 mg/ml dextran (middle panel) or 5 mg/ml recombinant human albumin (bottom panel) for 8 hrs. Cells were stained with an antibody against NFκB and with DAPI for nuclear localization. Albumin treatment, but not dextran treatment, was associated with translocation of NFκB to the cell nucleus. *B*, Albumin exposure leads to IκB degradation in podocytes. Western blot of lysate from podocytes treated with 5 mg/ml recombinant human albumin (A) or dextran (D) for 1 hr, 3 hrs or 6 hrs and probed with an antibody to IκB. Albumin treatment causes an initial degradation of IκB which is required for NFκB activation. α-tubulin is shown as a loading control. Graph to the right of the Western shows densitometric quantitation of the IκB band normalized to α-tubulin. * denotes P = 0.03 compared to dextran treated control cells.

IL-6 and TNF have been shown to activate NFκB [24], [25]. NFκB, is sequestered in an inactive form by IκB in the cytoplasm. Degradation of IκB yields translocation of NFκB to the cell nucleus and activation of gene transcription [25]. Since both IL-6 and TNF were significantly increased in podocytes exposed to albumin we hypothesized that albumin exposure would result in NFκB activation. As shown in Figure 5A, while dextran treated cells displayed cytoplasmic NFκB localization, albumin treatment yielded translocation of NFκB to the nucleus, indicating NFκB activation. To further confirm this finding we assessed IκB protein levels by western blot (Fig. 5 B and C). As expected, IκB protein levels were significantly lower in albumin treated cells compared to dextran treated controls (P = 0.03), indicating IκB degradation and supporting NFκB activation.

### Albumin Overload in vivo Induces Proteinuria and Glomerular Inflammatory Reponse

**Figure 6:**
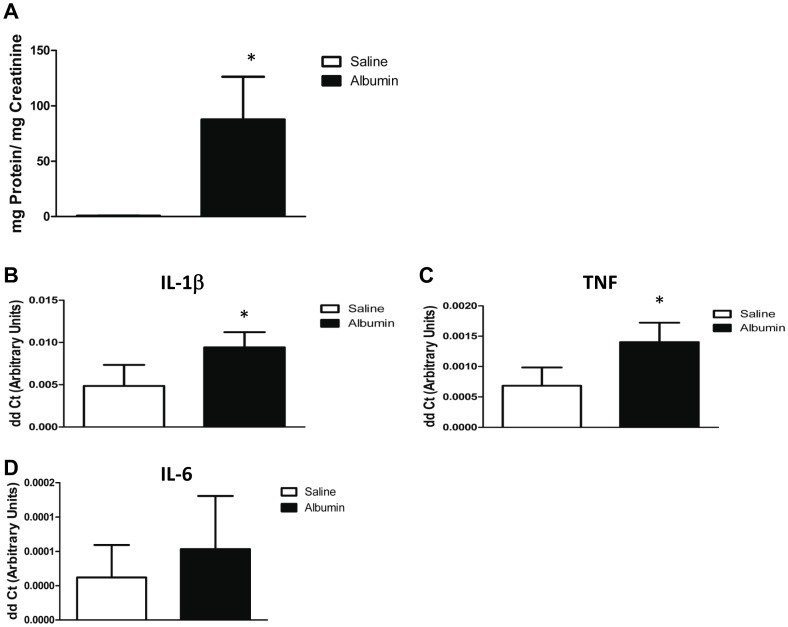
Albumin overload induces proteinuria and upregulates pro-inflammatory cytokine expression in isolated mouse glomeruli. *A*, Urinary protein normalized to urinary creatinine in mice injected with saline (open bar) or low endotoxin bovine serum albumin (BSA; closed bar; n = 4 animals per group). * denotes P = 0.004 compared to saline injected controls. *B*, IL-1β expression in isolated glomeruli from mice injected with saline (open bar) or BSA (closed bar; n = 4 animals per group). * denotes P = 0.02 compared to controls. *C*, TNF expression in isolated glomeruli from mice injected with saline (open bar) or BSA (closed bar; n = 4 animals per group). * denotes P = 0.02 compared to controls. *D*, IL-6 expression in isolated glomeruli from mice injected with saline (open bar) or BSA (closed bar; n = 4 animals per group).

To confirm these findings in an *in vivo* model of nephrotic syndrome the mouse albumin overload model was used. This model is preferable to those that use toxins such as adriamycin or lipopolysaccharide to induce albuminuria as it allows an evaluation of the direct effects of increased albumin transit through the glomerulus without the confounding effects of a podocyte poison. As shown in Figure 6A, mice injected with BSA developed significant proteinuria after 4 days of albumin injection, whereas saline injected mice displayed almost no proteinuria (Fig. 6A, P = 0.004). After 6 sequential BSA injections, glomeruli isolated from albumin-injected mice had a significant increase in IL-1β and TNF RNA expression compared to saline injected animals, P = 0.02; Fig. 6B and 6C). Expression of IL-6 was very low in the glomeruli of both saline and albumin injected animals making an accurate comparison of differences in the expression level of this cytokine difficult, although there was a trend towards increased IL-6 expression in glomeruli isolated from BSA-injected mice (Fig. 6D).

## Discussion

Podocytes contain increased amounts of albumin in kidney biopsies obtained from patients with nephrotic syndrome as well as in animals with heavy proteinuria [13], [26]. There is controversy however as to whether prolonged exposure to albumin is deleterious to podocytes. Here we show in a cultured human podocyte line that exposure to levels of albumin similar to what might be seen in the glomerular ultrafiltrate of patients with nephrotic syndrome increases cell death, up-regulates pro-apoptotic pathways and up-regulates inflammatory cytokines. We do not believe that these findings are due to contamination of the albumin with endotoxin, free fatty acids or globulins. In our studies we used recombinant human albumin which is fatty acid and globulin free and independently tested our albumin preparation to ensure that the levels of endotoxin present were extremely low (essentially endotoxin free by industry standards).

To examine the effects of albumin on podocytes *in vivo* we used a mouse protein overload nephropathy model. Glomeruli isolated from mice injected with BSA had a significant increase in TNF and IL-1β expression, lending support to our *in vitro* observations. Of interest is that glomerular expression of IL-6 was very low in both saline and BSA-injected animals. This may be due to the fact peak IL-6 expression occurs prior to day 9 post injection or to the fact that the pattern of pro-inflammatory cytokine expression differs somewhat between the *in vivo* and *in vitro* model.

We chose to use a recently described podocyte line for these studies [17]. Upon differentiation, these cells express WT-1, synaptopodin and nestin proteins. On the other hand, they lack important features of fully differentiated podocytes, including expression of nephrin and podocin proteins, at least when cultured under standard conditions. Because these cells can be isolated from human urine they allow for characterization of basic biologic processes within podocytes from subjects with normal renal function and those with glomerular diseases. Studies to examine whether podocytes isolated from healthy controls respond differently to albumin compared to those from patients with glomerular diseases are currently underway.

We also confirmed some of our key findings in a previously characterized human podocyte line that was initially isolated from a human nephrectomy specimen by Saleem et al [19]. We found that, as with the podocyte line isolated from human urine, albumin caused a dose dependent increase in cell death (Figure S1) and also increased production of IL-1β, TNF and IL-6 (Figure S2) in podocytes isolated from the nephrectomy specimen.

While previous work has shown that exposure to albumin in cultured podocytes induces endoplasmic reticulum stress, up-regulates TGF-β and increases apoptosis, these studies used relatively high doses of albumin (10–40 mg/ml) and did not control for the oncotic effects of albumin. In addition, these studies used bovine serum albumin, did not quantitate the amount of endotoxin present and examined the effects of albumin on mouse and not human podocytes [15], [16], [27]. In our work we used 5 mg/ml albumin (a dose similar to the concentration of albumin found in the urine of patients with nephrotic syndrome). As an oncotic control we used dextran of a similar molecular mass to albumin. Our studies used recombinant human albumin which is fatty acid and globulin free. We also independently verified that the concentration of endotoxin in this preparation is 0.005 EU endotoxin/mg albumin (essentially endotoxin free by industry standards). In addition, we are the first to report that albumin exposure up-regulates pro-inflammatory cytokine production by podocytes both *in vitro* and *in vivo*.

The mechanisms underlying albumin uptake in podocytes remain to be determined. Previous work has demonstrated that albumin endocytosis in podocytes is a receptor mediated process and that the receptors involved have some specificity for albumin [14]. In proximal tubule cells the multiligand receptors megalin and cubilin are required for albumin endocytosis [28], [29]. Human podocytes have recently been shown to express both megalin and cubilin [30], [31] but their role in podocyte albumin uptake remains unknown.

It is also unclear at present precisely how albumin induces a pro-apoptotic and inflammatory cascade in cultured podocytes. In proximal tubule cells, albumin has been shown to activate the renin angiotensin system and NFκB via a protein kinase C, NADPH oxidase dependent pathway [32], [33]. Albumin has also been shown to induce reactive oxygen species production and interleukin-8 production in renal proximal tubule cells via a protein kinase C dependent pathway [34]. Albumin overload also up-regulates monocyte chemoattractant protein-1 in proximal tubules via the extracellular signal-regulated (ERK1/2) and mitogen-activated protein kinase (MAPK) pathways [35].

In conclusion, we have found that albumin exposure at levels comparable to what is found in the urine of patients with nephrotic syndrome increased cell death, upregulated pro-inflammatory cytokines and upregulated pro-apoptotic pathways in a cultured podocyte-like cell line. In addition, glomeruli isolated from mice with nephrotic syndrome also had increased expression of pro-inflammatory cytokines. Podocytes are terminally differentiated cells with limited regenerative capability [36]. Our findings support the notion that prolonged albumin exposure is deleterious to podocytes and may contribute to the progressive podocyte loss seen in proteinuric kidney disease. Further studies might address the mechanisms underlying induction of inflammatory and pro-apoptotic cascades by albumin.

## Supporting Information

Figure S1 **Albumin exposure increases cell death in cultured human podocytes isolated by Saleem et al.** *A*, Podocytes were treated with low endotoxin recombinant human albumin (closed bars) or dextran (open bars) and cell death at 24 hrs was measured using the trypan blue exclusion assay. *denotes P = 0.001. *B*, Cell death after treatment of podocytes with human albumin for 48 hrs. * denotes P = 0.0001.
(TIF)

Figure S2 **Albumin exposure increases pro-inflammatory cytokine release in cultured human podocytes isolated by Saleem et al.** *A*, Amount of IL-1β normalized to total cellular protein produced by podocytes after treatment with 5 mg/ml recombinant human albumin (closed bars) or 5 mg/ml dextran (open bars) for varying amounts of time. * denotes P<0.0001 compared to dextran treated controls. *B*, Amount of TNF normalized to total cellular protein released into the medium by podocytes after treatment with albumin (closed bars) or dextran (open bars) for varying amounts of time. * denotes P = 0.006 compared to dextran treated control cells. *C*, Levels of IL-6 normalized to total cellular protein released into the medium by podocytes treated with albumin (closed bars) or dextran (open bars) for varying amounts of time. * denotes P = 0.0009 compared to dextran treated controls.
(TIF)
