# Supplementary figures and images for: Endocytosis of Albumin by Podocytes Elicits an Inflammatory Response and Induces Apoptotic Cell Death
Source: PLoS One. 2013 Jan 28;8(1):e54817. doi: 10.1371/journal.pone.0054817 (PMC3557279; doi:10.1371/journal.pone.0054817)

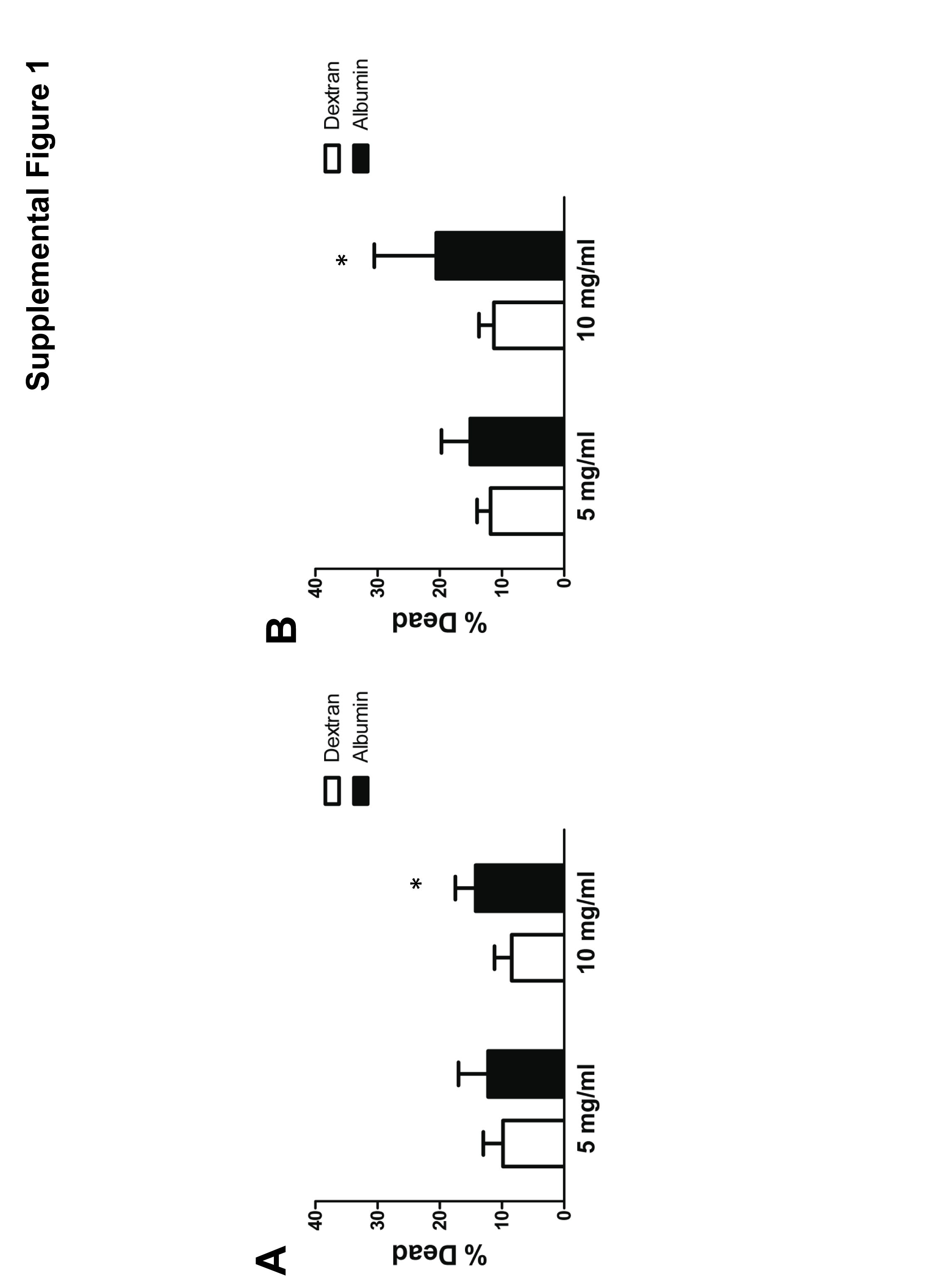

Supplement: Figure S1 — Albumin exposure increases cell death in cultured human podocytes isolated by Saleem et al. A, Podocytes were treated with low endotoxin recombinant human albumin (closed bars) or dextran (open bars) and cell death at 24 hrs was measured using the trypan blue exclusion assay. *denotes P = 0.001. B, Cell death after treatment of podocytes with human albumin for 48 hrs. * denotes P = 0.0001. (TIF) [file pone.0054817.s001.tif]

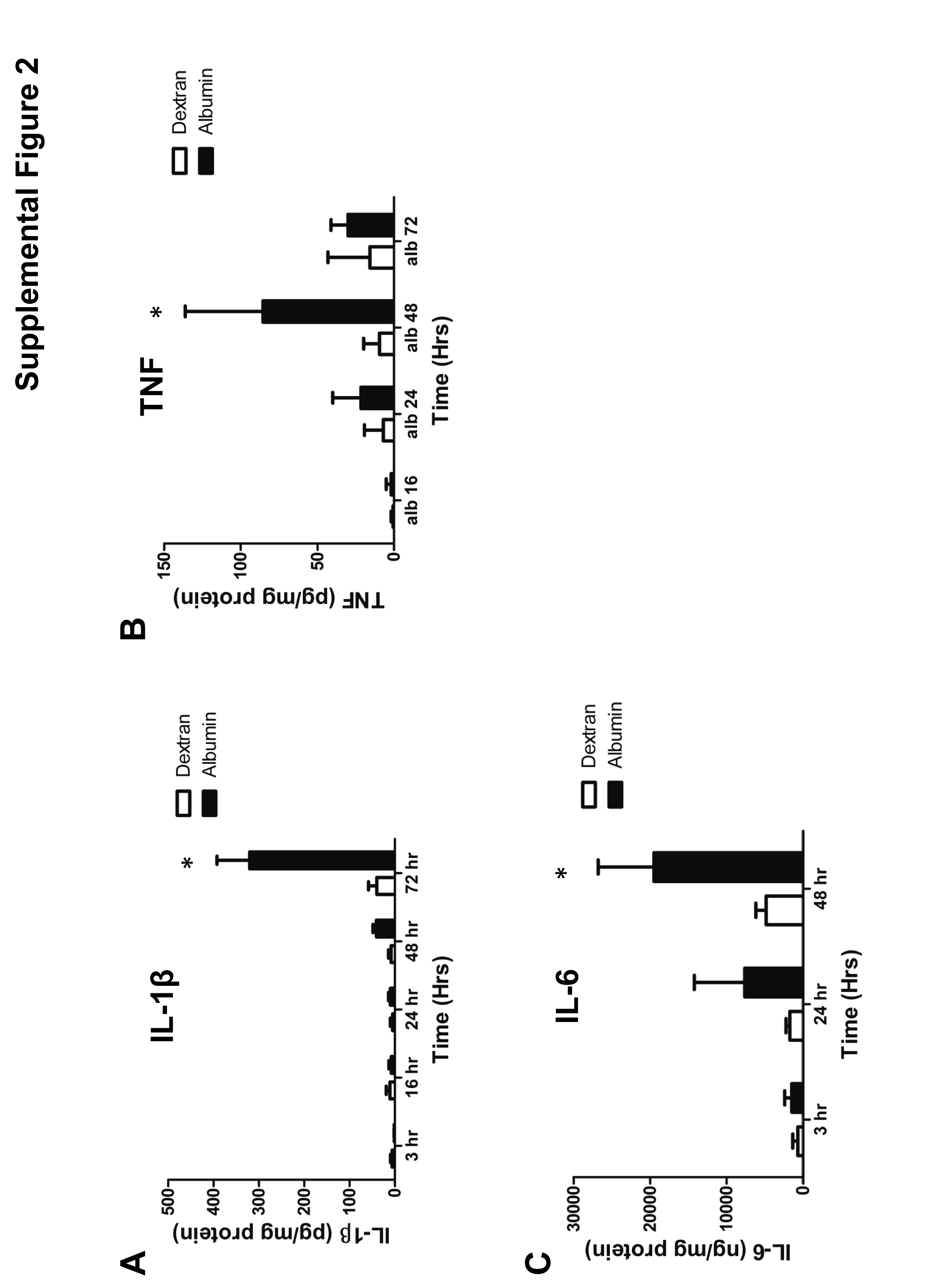

Supplement: Figure S2 — Albumin exposure increases pro-inflammatory cytokine release in cultured human podocytes isolated by Saleem et al. A, Amount of IL-1β normalized to total cellular protein produced by podocytes after treatment with 5 mg/ml recombinant human albumin (closed bars) or 5 mg/ml dextran (open bars) for varying amounts of time. * denotes P<0.0001 compared to dextran treated controls. B, Amount of TNF normalized to total cellular protein released into the medium by podocytes after treatment with albumin (closed bars) or dextran (open bars) for varying amounts of time. * denotes P = 0.006 compared to dextran treated control cells. C, Levels of IL-6 normalized to total cellular protein released into the medium by podocytes treated with albumin (closed bars) or dextran (open bars) for varying amounts of time. * denotes P = 0.0009 compared to dextran treated controls. (TIF) [file pone.0054817.s002.tif]
